# From Diagnosis to Intervention: Targeting Rate of Force Development in Locomotive Syndrome

**DOI:** 10.1111/ggi.70361

**Published:** 2026-01-20

**Authors:** I‐Ling Chen, Chao‐Chun Huang

**Affiliations:** ^1^ Department of Physical Therapy Far Eastern Memorial Hospital New Taipei City Taiwan; ^2^ Department of Physical Medicine and Rehabilitation Far Eastern Memorial Hospital New Taipei City Taiwan; ^3^ Department of Physical Therapy and Assistive Technology National Yang Ming Chiao Tung University Taipei Taiwan

To the Editor,

We read with great interest the recent study by Seya et al. [1] regarding the association between sit‐to‐stand (STS) biomechanics and Locomotive Syndrome (LS) severity. The authors are to be commended for identifying that the rate of force development (RFD/w), rather than maximal strength (F/w), acts as the sole independent predictor of LS stage (OR: 0.84, *p* = 0.002). This finding aligns with the “dynapenia” hypothesis, suggesting that deficits in rapid neural drive often precede the loss of absolute muscle mass in middle‐aged and older adults.

Two methodological points warrant further discussion to contextualize these findings for clinical application.

First, regarding the use of the zaRitz BM‐220 system (80 Hz), while laboratory‐based assessments of early RFD (e.g., 0–50 ms) typically employ sampling frequencies ≥ 1000 Hz to capture the steep slope of neural discharge [2], device‐based STS systems are designed to balance precision with clinical feasibility. Consequently, the reported RFD values likely reflect a functional average rather than the maximal explosive capacity seen in high‐frequency settings. Recognizing this distinction is important when comparing these clinical findings with physiological literature, suggesting that future validation studies may be needed to confirm sensitivity to subtle post‐intervention changes.

Second, identifying RFD as a biomarker highlights a critical opportunity for intervention design. Traditional power training—essential for improving RFD—often involves high‐velocity movements that generate significant eccentric braking forces. For patients with LS, who may have comorbidities such as osteoporosis or osteoarthritis, this presents a safety challenge. In this context, “concentric‐only” resistance strategies offer a viable solution. Our previous randomized controlled trial demonstrated that hydraulic resistance training, which eliminates eccentric load, significantly improved contractile RFD (0–200 ms) by approximately 27% in patients with musculoskeletal comorbidities [3]. Unlike weight‐based isotonic training, hydraulic mechanisms allow patients to exert maximal acceleration without the risk of impact during the deceleration phase.

Seya et al. have successfully established the diagnostic necessity of RFD. One potential next step for the geriatric rehabilitation community may be the exploration of intervention protocols—such as hydraulic or pneumatic power training—that can safely target this specific neuromuscular deficit while minimizing injury risk in vulnerable populations.

## Funding

The authors have nothing to report.

## Disclosure

The authors have nothing to report.

## Ethics Statement

The authors have nothing to report.

## Consent

The authors have nothing to report.

## Conflicts of Interest

The authors declare no conflicts of interest.

## Data Availability

Data sharing is not applicable to this article as no new data were created or analyzed in this study.
